# The inactive X chromosome accumulates widespread epigenetic variability with age

**DOI:** 10.1186/s13148-023-01549-y

**Published:** 2023-08-25

**Authors:** Yunfeng Liu, Lucy Sinke, Thomas H. Jonkman, Roderick C. Slieker, BIOS Consortium, Erik W. van Zwet, Lucia Daxinger, Bastiaan T. Heijmans

**Affiliations:** 1 Molecular Epidemiology, Department of Biomedical Data Sciences, Leiden University Medical Center, Postzone S-5-P, 2333 ZC Leiden, The Netherlands; 2 Department of Cell and Chemical Biology, Leiden University Medical Center, 2333 ZC Leiden, The Netherlands; 3 Medical Statistics, Department of Biomedical Data Sciences, Leiden University Medical Center, 2333 ZC Leiden, The Netherlands; 4 Department of Human Genetics, Leiden University Medical Center, 2333 ZC Leiden, The Netherlands

**Keywords:** DNA methylation, Aging, X chromosome, Women, Variance, Gene expression

## Abstract

**Background:**

Loss of epigenetic control is a hallmark of aging. Among the most prominent roles of epigenetic mechanisms is the inactivation of one of two copies of the X chromosome in females through DNA methylation. Hence, age-related disruption of X-chromosome inactivation (XCI) may contribute to the aging process in women.

**Methods:**

We analyzed 9,777 CpGs on the X chromosome in whole blood samples from 2343 females and 1688 males (Illumina 450k methylation array) and replicated findings in duplicate using one whole blood and one purified monocyte data set (in total, 991/924 females/males). We used double generalized linear models to detect age-related differentially methylated CpGs (aDMCs), whose mean methylation level differs with age, and age-related variably methylated CpGs (aVMCs), whose methylation level becomes more variable with age.

**Results:**

In females, aDMCs were relatively uncommon (*n* = 33) and preferentially occurred in regions known to escape XCI. In contrast, many CpGs (*n* = 987) were found to display an increased variance with age (aVMCs). Of note, the replication rate of aVMCs was also high in purified monocytes (94%), indicating an independence of cell composition. aVMCs accumulated in CpG islands and regions subject to XCI suggesting that they stemmed from the inactive X. In males, carrying an active copy of the X chromosome only, aDMCs (*n* = 316) were primarily driven by cell composition, while aVMCs replicated well (95%) but were infrequent (*n* = 37).

**Conclusions:**

Our results imply that age-related DNA methylation differences at the inactive X chromosome are dominated by the accumulation of variability.

**Supplementary Information:**

The online version contains supplementary material available at 10.1186/s13148-023-01549-y.

## Background

Epigenetic alterations are one of the five primary hallmarks of aging [1]. A primary role for epigenetic mechanisms is the inactivation of one copy of the X chromosome in females to maintain dosage equivalence between females who carry two copies of the X chromosome, and males who carry a single copy [2]. Since the X chromosome harbors hundreds of protein-coding genes, many of which are implicated in disease including cancer [3] and neurological diseases [4, 5], age-related epigenetic changes at the inactivated X chromosome may be relevant for female aging.

DNA methylation is tightly involved in the process of X-chromosome inactivation (XCI) [6]. However, the impact of age on the methylation of chromosome X in females remains unclear. Three recent studies addressed this question in whole blood samples and reported sets of CpG dinucleotides whose methylation level was associated with age separately for males and females [7, 8] or showed statistical evidence for an interaction between age and sex [9]. However, there was a striking lack of overlap between the results of these studies. Moreover, the previous studies focused on the occurrence of differences in mean methylation with age [7–9] while there is increasing attention for the accumulation of variability in DNA methylation with age, a phenomenon that appears to be relatively independent of cell composition changes with age [10–13]. Finally, previous analyses did not investigate whether CpGs affected by age-related differences in DNA methylation in females mapped to regions subject to XCI. This is important because approximately 75% of genes on the inactive X are subject to XCI, while 15% of genes on the inactive X consistently escape XCI and the escape status of an additional 10% of genes varies between tissues and individuals [14–16]. Hence, a substantial proportion of age-related DNA methylation observed in females may not occur in regions subject to XCI and presumably are independent of X-inactivation. These outstanding questions may be solved by analyzing larger sample numbers with robust statistical methods to test both differences in mean and variance followed by in-depth genomic annotation to relate findings to XCI.

Here, we report on the analysis of multiple discovery and replication cohorts totaling 3,334 female and 2,612 male blood samples with methylation data on 9,777 CpGs mapping to the X chromosome as obtained using the Illumina 450k methylation array. We detected and replicated age-related differentially methylated CpGs (aDMCs), whose mean methylation level differs with age, and age-related variably methylated CpGs (aVMCs), whose methylation level becomes more variable with age, while accounting for the impact of blood cell composition and inflation of test statistics. Systematic annotation, interpretation, and integration with transcriptomics data indicate that the inactive X is primarily affected by the accumulation of variance in DNA methylation with age, while the differences in mean are common at the active X chromosome but depend on changes in cell counts with age.

## Methods

### Discovery cohorts

To discover age-related differentially methylated CpGs (aDMCs) and age-related variably methylated CpGs (aVMCs), genome-wide DNA methylation data were generated in whole blood samples within the Biobank-based Integrative Omics Studies (BIOS) Consortium, which comprises six Dutch biobanks: Cohort on Diabetes and Atherosclerosis Maastricht (CODAM) [17], LifeLines (LL) [18], Leiden Longevity Study (LLS) [19], Netherlands Twin Register (NTR) [20], Rotterdam Study (RS) [21], and the Prospective ALS Study Netherlands (PAN) [22]. Discovery data used in this study consist of 4031 (2343/1688 females/males) unrelated individuals for which DNA methylation data were available (Additional file 1: Table S1). For 3131 (1794/1337 females/males) of these individuals also RNA-seq data were available (Additional file 1: Table S1). Data linkage of the two data types was verified from genotype data using *OmicsPrint* [23]. In addition, data on age, sex and technical batches were available for each cohort.

The generation of DNA methylation data has been described previously [24]. In brief, 500 ng of genomic DNA was bisulfite converted by the EZ DNA Methylation kit (Zymo Research, Irvine, CA, USA), and 4 μl of bisulfite-converted DNA was measured on the Illumina HumanMethylation450 array using the manufacturer’s protocol (Illumina, San Diego, CA, USA). Preprocessing and normalization of the data were done using DNAmArray workflow previously developed by our group (https://molepi.github.io/DNAmArray_workflow/). First, original IDAT files were imported into by R package *minfi* [25], followed by sample-level quality control (QC) was performed using *MethylAid* [26]. Filtering of probes was based on detection *P* value (*P* < 0.01), number of beads available (≤ 2), or zero values for signal intensity. Normalization was done using functional normalization as implemented in *minfi* [25], using five principal components extracted using the control probes for normalization. All samples or probes with more than 5% missing were excluded. In addition, probes with ambiguously mapping or cross-reactive were removed [27]. Finally, 9,777 X-chromosome CpGs and 4,031 samples were included in discovery set. Prior to analysis, we used *Combat* function of the *SVA* package to remove residual batch effects between cohorts, with biobank as batch and age, sex and known technical batches as covariates [28]. To exclude a negative impact of non-normal distributions and outliers on the validity of our results, DNA methylation data were transformed by rank-inverse normal (RIN) transformation for each cohort and females and males separately [29, 30].

Detailed information on the generation and processing of the RNA-seq data can be found in previous work [31]. In short, globin transcripts were removed from whole blood RNA using the Ambion GLOBINclear kit and subsequently processed for RNA-sequencing using the Illumina TruSeq version 2 library preparation kit. RNA libraries were paired-end sequenced using Illumina’s HiSeq 2000 platform with a read length of 2 × 50 bp, pooling 10 samples per lane. Reads which passed the chastity filter were extracted with CASAVA. Quality control was done in three steps: initial QC was performed using FastQC (v0.10.1), adaptor sequences were removed using Cutadapt, and read ends with insufficient quality were removed with Sickle. Reads were aligned to the human genome (hg19) using STAR (v2.3.0e). To avoid reference mapping bias, all GoNL SNPs (http://www.nlgenome.nl/) with MAF > 0.01 in the reference genome were masked with N. Read pairs with at most 8 mismatches, mapping to at most 5 positions, were used. Gene counts were calculated by summing the total number of reads aligning to a gene’s exons according to Ensembl, version 71. Samples for which less than 70% of all reads mapped to exons were removed.

For this study, we analyzed protein-coding genes mapping to the X chromosome. Raw counts of genes were transformed to log counts per million (CPM) values. After filtering out lowly expressed genes from the dataset (median CPM < 1), 512 out of 830 genes remained and were used for further analysis. Similar to DNA methylation data, RNA-seq data were transformed by rank-inverse normal (RIN) transformation for each cohort and females and males separately [29, 30].

### Replication cohorts

Two external DNA methylation datasets were used to replicate our results: a whole blood dataset originated from Sweden Population Health study by Johansson et al. [32] and a purified monocytes dataset originated from Multi-Ethnic Study of Atherosclerosis by Reynolds et al. [33]. These datasets here were referred to as Johansson Blood and Reynolds Monocytes. Detailed information is shown in Additional file 1: Table S1.

For the Johansson Blood dataset, raw IDATA files were downloaded from the Gene Expression Omnibus (GEO) database (GSE87571). Also, information on age and sex for was available from GEO for this data set. The processing and normalization procedure of DNA methylation data is same as above. After processing, 9,853 X-chromosome CpGs were available. For the Reynolds Monocytes dataset, DNA methylation array data were available from GEO (GSE56046) as quantile normalized data obtained using the *lumi* package [34]. The GEO accession also included data on age and purity data of isolated monocytes. The sex of samples was predicted by *DNAmArray* package. After quality control, 9,861 X-chromosome CpGs remained. Both replication cohorts included all 9,777 X-chromosome CpGs measured in the discovery cohort.

### Detecting aDMCs and aVMCs on X-chromosome

To detect DNA methylation differences in both mean (aDMCs) and variance (aVMCs) with age, we applied double generalized linear model (DGLM). DGLM is a fully parametric method that first estimates mean effects by the linear model and then variance effects by the dispersion sub-model [35]. DGLM iterates between models until convergence. We used DGLM as implemented in the *dglm* R package (https://cran.r-project.org/web/packages/dglm/index.html) to identify aDMCs and aVMCs on chromosome X separately for females and males. The mean model part of DGLM was used to identify aDMCs, correcting for known covariates (age, cohort, cell counts and technical batches, namely sentrix position and sample plate) and unknown covariates include 5 latent factors estimated by *SVA* package [28]. Blood cell composition was estimated using the IDOL method as implemented in the R package *minfi* [25] and resulted in predicted fractions for CD8T cells, CD4T cells, NK cells, B cells, monocytes and granulocytes. Of note, granulocytes were excluded from the model to exclude collinearity so that the effect of this cell type becomes included in the intercept.

1 $$\begin{aligned} DNAm_{i} & = \beta_{0} + \beta_{1} *Age + \beta_{2} *Biobank + \beta_{3} *CD8T\% + \beta_{4} *CD4T\% \\ & \quad + \beta_{5} *NK\% + \beta_{6} *Bcell\% + \beta_{7} *Monocyte\% \\ & \quad + \beta_{8} *Sentrix\,position + \beta_{9} *Sample\,plate + \beta_{j} *Latent\,factor1 \ldots 5 + \varepsilon_{i} \\ \end{aligned}$$

where ${DNAm}_{i}$ represent 9,777 X-chromosome CpGs methylation matrix, $Biobank$ represent 6 cohorts comprising the data, $Sentrix\,position$ represent sample position on the 450K array, $Sample\,plate$ represent bisulfite plate and ${\beta }_{j}$ represent regression coefficient for 5 latent factors estimated using *SVA* package [28], and ${\varepsilon }_{i}$ represents the residual. Age-related differentially methylation changes were assessed by the parameter ${\beta }_{1}$ in the mean model.

Then we estimated variance effect of age by the parameter ${\gamma }_{1}$ in the dispersion sub-model:

2 $$\varepsilon_{i} \sim N\left( {0,\sigma^{2} \exp \left( {\gamma_{1} *Age + \gamma_{2} *Biobank + \gamma_{3} *CD8T\% + \gamma_{4} *CD4T\% + \gamma_{5} *NK\% + \gamma_{6} *Bcell\% + \gamma_{7} *Monocyte\% + \gamma_{8} *Sentrix\,position + \gamma_{9} *Sample\,plate + \gamma_{j} *Latent\,factor1 \ldots 5} \right)} \right)$$

To reduce false positive findings, a Bayesian method implemented in R package *bacon* was used to correct the bias and inflation of test-statistics generated from mean model and dispersion sub-model of DGLM [36]. Statistically significance of the CpGs was determined by correcting multiple testing with a Bonferroni corrected *P* value < 0.05.

To replicate findings, we repeated sex-stratified analysis with the same statistical model in Johansson Blood and Reynolds Monocytes datasets separately for all CpGs that were detected as aDMCs or aVMCs in the discovery data set. For Johansson Blood data, cell counts were predicted the same way as for the discovery cohort. For the Reynolds Monocytes data, cell purity was included as covariate. CpGs were considered replicated if significant after correcting for multiple testing using the false discovery rate (FDR) (*P*_FDR_ < 0.05) [37] and the direction of the effect was the same in both replication cohorts. The number of tests considered was the total number of unique aDMCs or aVMCs detected in the discovery analysis (i.e., males + female).

To assess the number of distinct genomic loci affected by aDMCs or aVMCs, differentially methylated regions (DMRs) and variably methylated regions (VMRs) were first called among replicated aDMCs and aVMCs in females and males separately by the *DMRfinder* algorithm [38] as implemented in the *DNAmarray* workflow (https://molepi.github.io/DNAmArray_workflow/). DMRs and VMRs were defined as regions with at least 3 differentially or variably methylated positions (DMCs/VMCs) with an inter-CpG distance ≤ 1 kb, allowing maximum of three non-DMCs/non-VMCs across a DMR/VMR [38]. Next, the number of distinct loci were calculated as the total number of aDMCs/aVMCs minus the number of aDMCs/aVMCs in DMRs/VMRs plus number of DMRs/VMRs called by *DMRfinder* [38].

### Genomic features of aDMCs and aVMCs

**Fig. 1:**
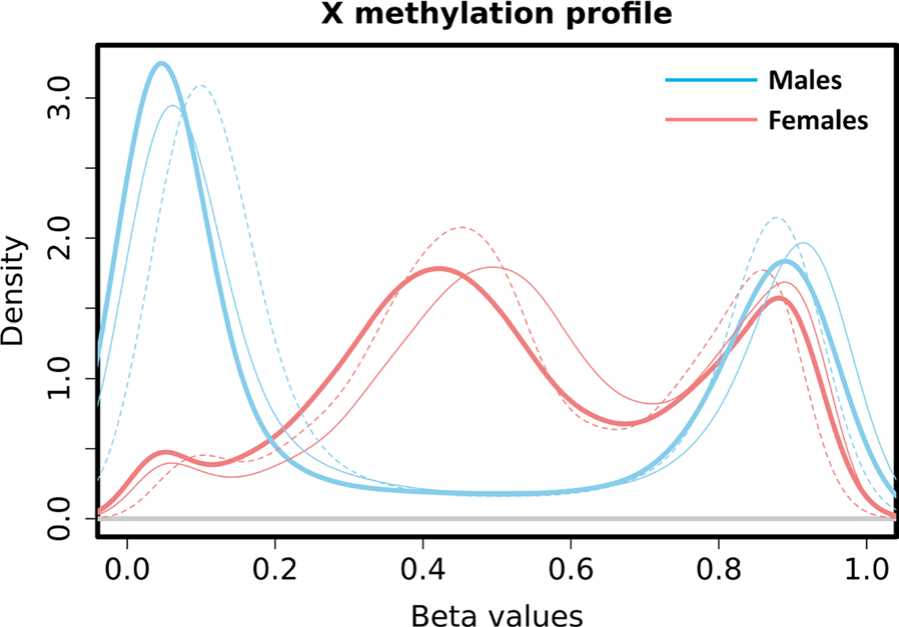
Distribution of X-methylation in females and males based on 9777 CpGs in the discovery data (thicker solid line: BIOS Blood) and replication data (thinner solid line: Johansson Blood; thinner dash line: Reynolds Monocytes). The blue bimodal line and red trimodal line represent the distribution of X-methylation in males and females, respectively

Replicated aDMCs and aVMCs were annotated according to 3 features. First, the CpGs involved were divided into three categories based on mean DNA methylation level: hypomethylated (*β* < 0.25), intermediately methylated (0.25 < *β* < 0.7) and hypermethylated (*β* > 0.7) since DNA methylation level is closely linked to XCI. Intermediate methylation is typical for XCI, where the Xa is hypomethylated and the Xi is hypermethylated and the measured methylation is the average of the two chromosomes. Hypomethylation is typical for regions escaping XCI, where both the Xa and Xi display low methylation levels. For hypermethylation, the XCI status remains undefined. The hypomethylated and hypermethylated thresholds were chosen based on the location of the hypo- and hyper-methylated peaks in the density plots of male X-methylation in BIOS discovery data (Fig. 1)*.* Second, CpGs were mapped to 3 CpG island-related features: CpG islands (CGIs), CGI shores and non-CGI regions, since XCI is associated with hypermethylation of CGIs [6]. The annotation was based on CGI-track downloaded from UCSC Genome Browser using version hg19 of the human genome. CGI shores were annotated as 2 kb regions flanking the CGIs and all remaining regions as non-CGI [38]. Finally, we annotated CpGs according to XCI status of the inactive X chromosome. Consensus XCI status calls per gene were previously defined [39] on the basis of three published studies [14, 15, 40] and resulted in genes classified into three categories: subject to XCI, escape XCI and variably escape XCI. These XCI status calls are referred to as meta-status calls [41]. For the annotation, we mapped CpGs to the nearest transcription start site (TSS) and when within 2kb from a TSS, the XCI status of the CpGs was set equal to the XCI status of the gene associated with the TSS. CpGs further away than 2kb from a TSS were not annotated because of uncertainty about their XCI status [42]. To test whether aDMCs and aVMCs were enriched for each of the three annotations (methylation level categories, CGI annotation, and XCI status), their distributions were compared with that of all X-chromosome CpGs that were not identified as aDMC or aVMC in males or females using a Chi-square test or a Fisher’s exact test (if expected cell counts were < 5).

### Associations with X chromosome gene expression levels

To explore potential functional consequences of aDMCs and aVMCs, a linear regression model using the R package *limma* [43] was fitted to test for associations between the CpGs involved and the expression of 512 protein-coding genes mapping to the X chromosome. For this analysis, 3131 samples (1794/1337 females/males) from the BIOS consortium with both DNA methylation and gene expression were used (Additional file 1: Table S1). Known covariates included age, cohort, white blood cell composition as estimated with the R package *minfi* [25] and technical batches (e.g., sentrix position, sample plate and flowcell number) were corrected in the linear regression model. Additionally, latent factors as estimated with the *SVA* package were included [28].

3 $$\begin{aligned} {Expression}_{i}& ={\delta }_{0}+ {\delta }_{1}*DNAm+{\delta }_{2}*Age+{\delta }_{3}*Biobank+{\delta }_{4}*CD8T\%+{\delta }_{5}*CD4T\%\\&\quad +{\delta }_{6}*NK\%+{\delta }_{7}*Bcell\%+{\delta }_{8}*Monocyte\%+{\delta }_{9}*Sentrix\,position+{\delta }_{10}*Sample\,plate\\&\quad +{\delta }_{11}*Flowcell\,Number+{\delta }_{j}*Latent\,factor 1\dots 5+ {\varepsilon }_{i} \\ \end{aligned}$$

where ${Expression}_{i}$ represent 512 X-chromosome genes expression matrix, $Biobank$ represent 6 cohorts comprising the data, $Flowcell\,Number$ represent the HiSeq 2000 flowcell used for RNA-seq measurement,${\delta }_{j}$ represent regression coefficient for 5 latent factors estimated by *SVA* package [28], and ${\varepsilon }_{i}$ represents the residual. We used the R package *bacon* [36] to correct bias and inflation in the test statistics generated by this linear regression model and follow by multiple testing correction using the Bonferroni method (*P*_*bonf*_ < 0.05). Specifically, the bias and inflation of t-statistics was corrected for associations between each CpG and 512 investigated X-linked genes.

For X-linked genes associated with at least 1 CpG, we performed Gene Ontology enrichment analysis using the R package *clusterProfiler* using all other X-linked genes as background [44].

## Results

### Identification and replication of aDMCs and aVMCs on X-chromosome

To uncover age-related methylation changes on the X chromosome, we analyzed the methylation of 9,777 CpGs in whole blood using a discovery data set of 2343 females and 1688 males followed by the replication of findings both in an external whole blood dataset (388/341 females/males; Table 1) and an external dataset based on purified monocytes (603/583 females/males; Table 1). As expected, X-chromosome methylation levels were distinct for females and males (Fig. 1). While the male X methylation pattern was similar to that of autosomes with the majority of CpGs being either hypo- or hypermethylated, the female X methylation pattern was trimodal with a significant proportion of CpGs showing intermediate DNA methylation levels. This stems from the fact that males carry a single active X, whereas females carry two copies of X, an active (Xa) and an inactive copy (Xi), where the Xi copy is predominantly hypermethylated as part of the mechanisms ensuring XCI.

**Table 1:** Number of age-related differentially methylated CpGs (aDMCs) in the discovery and replication stage

	Males			Females		
	*N*	aDMCs (loss/gain)	Replication rate	*N*	aDMCs (loss/gain)	Replication rate
*Discovery*						
BIOS Blood	1688	1837 (718/1119)	–	2343	80 (41/39)	–
*Replication*						
Johansson Blood	341	1371 (532/839)	75%	388	79 (41/38)	99%
Reynolds Monocytes	583	340 (195/145)	25%	603	34 (24/10)	43%
Doubly replicated	–	316 (175/141)	17%	–	33 (24/9)	41%

**Fig. 2:**
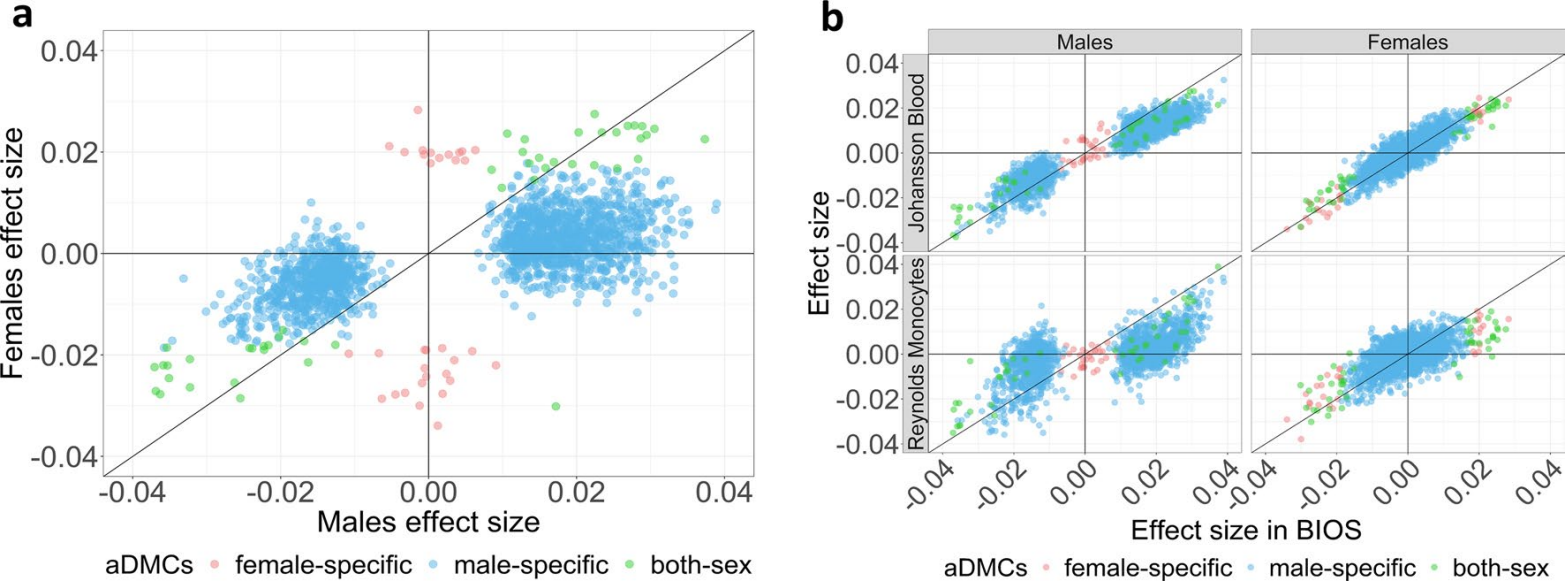
**a** Scatter plot of effect sizes for aDMCs observed in males and females in the discovery data set (BIOS Blood). Mean effect size of male-specific aDMCs in females were 0.3 times lower than that in males.** b** Scatter plot of aDMCs effect sizes in the discovery data sets and the Johansson Blood and Reynolds Monocytes replication datasets. Female-specific aDMCs, male-specific aDMCs and aDMCs statistically significant in both sexes are colored by red, blue and green, respectively. *aDMCs*—age-related differentially methylated CpGs

**Fig. 3:**
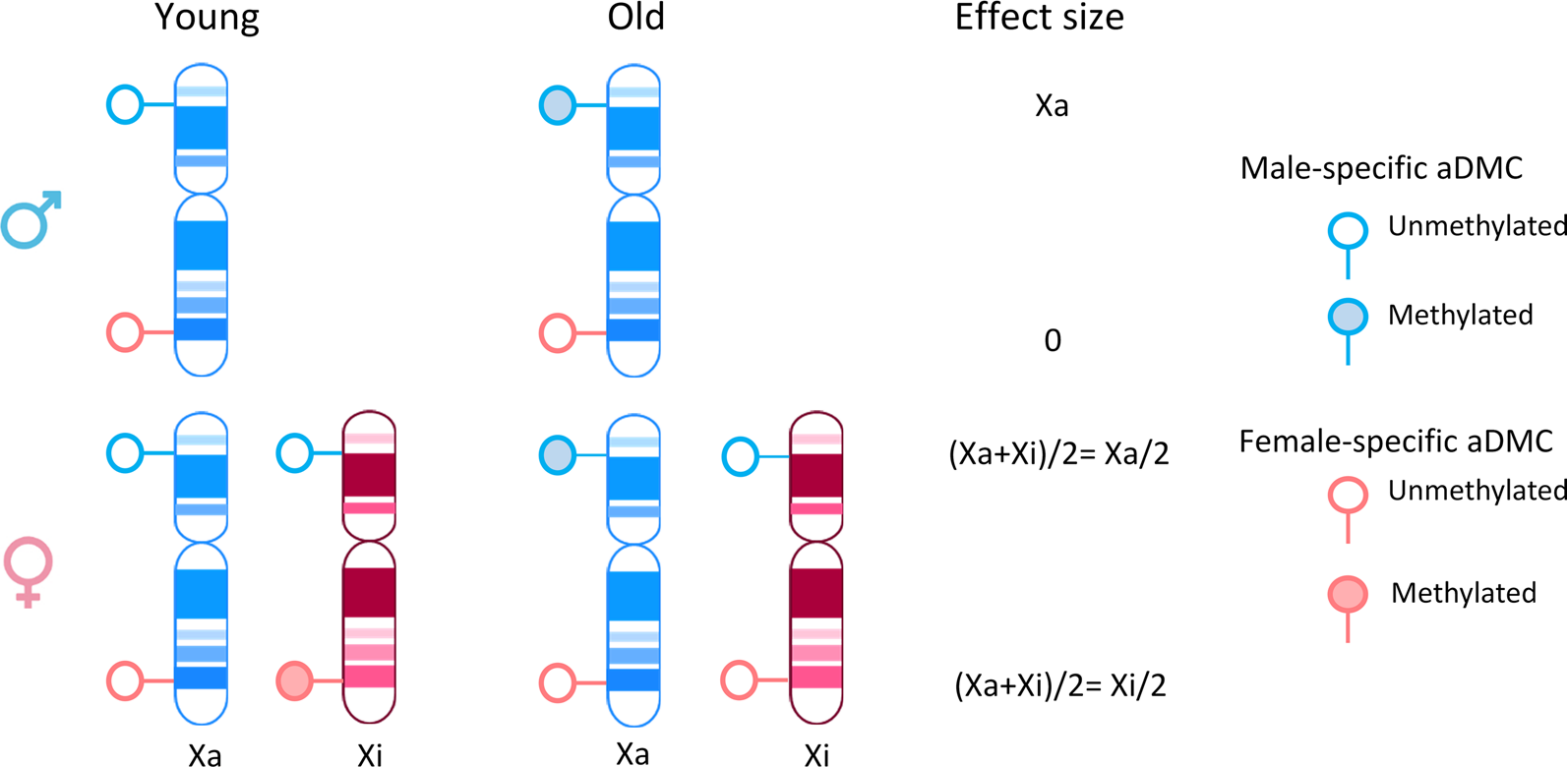
Schematic representation of how carrying a single copy of Xa only (males) or a copy of both Xa and Xi (females) affects the effect size of age-related DNA methylation differences observed in population studies. Note that the effect size of an aDMC specific for the Xa is twice that in males than females resulting in higher statistical power to detect such Xa-specific aDMCs in males than females. The same reasoning applies to aVMCs. Male-specific aDMC and female-specific aDMC are colored by blue and red, respectively. An empty circle indicates an unmethylated CpG, and a shaded circle a methylated CpG

We first focused on the detection of CpGs whose mean methylation differed with age (aDMCs) using DGLM. To confirm the robustness of this approach, we compared results with those obtained using a conventional model fitted using *limma* [40] and the effect sizes were virtually identical (Additional file 1: Fig. S1). In the discovery data set, we identified 1837 aDMCs in males but only 80 in females (P_*bonf*_ < 0.05; Table 1 and for a full list: Additional file 2: Tables S3 and S4). Of the aDMCs, 47 were shared between males and females. When inspecting the effect sizes for aDMCs, a striking pattern emerged (Fig. 2a): for female-specific aDMCs, the effect size in males was close to 0, whereas for male-specific aDMCs, the effect size in females was lower than in males, but correlated with that in males. This observation may be explained by the fact that males lack an Xi and, hence, female-specific aDMCs involving the Xi are fully absent in males (Fig. 3). In contrast, male-specific aDMCs involve the Xa only, and females also carry a copy of Xa. Hence, the male-specific aDMCs are expected to be also present in females, albeit diluted by the presence of the unaffected Xi copy. This effect would be expected to dilute the effect size two times (Fig. 3). Indeed, we observed that the effect size for male-specific aDMCs was on average reduced by a factor of 0.3 in females (Fig. 2a). In addition, we observed that the standard error of the effect sizes for these aDMCs in females were higher than in males (Additional file 1: Fig. S2). This may be explained by extra noise in the data for females due to the presence of a copy of Xi that does not affect Xa-specific aDMCs in males. The reduced effect size and greater standard error would together result in a reduced statistical power to detect aDMCs affecting Xa in females.

To replicate our findings, we used two external datasets based of whole blood and purified monocytes samples (Fig. 2b, Table 1). We found that aDMCs effect sizes observed in discovery cohort were consistent with the effect size in Johannsson Blood dataset resulting in replication rates of 75% and 99% for male and female aDMCs, respectively. However, in the Reynolds data set based on purified monocytes, the replication rate of male aDMCs reduced to 25%, suggesting that the occurrence of aDMCs on Xa as observed in whole blood is either only present in blood cell types other than monocytes or, which we consider more likely, depends on age-related changes in blood cell composition that is not captured by the  6 predicted cell types obtained using the IDOL method. For females aDMCs, the replication rate was somewhat higher at 43%. The number of aDMCs replicating in both data sets were 33 in females (none of which occurred in regions and hence all represented distinct loci) and 316 in males (distributed across 242 distinct loci; Table 1, Additional file 1: Fig. S3, Additional file 2: Table S3 and Table S4). Examples of the relationship between age and DNA methylation for replicated aDMCs are shown in Additional file 1: Fig. S4.

**Fig. 4:**
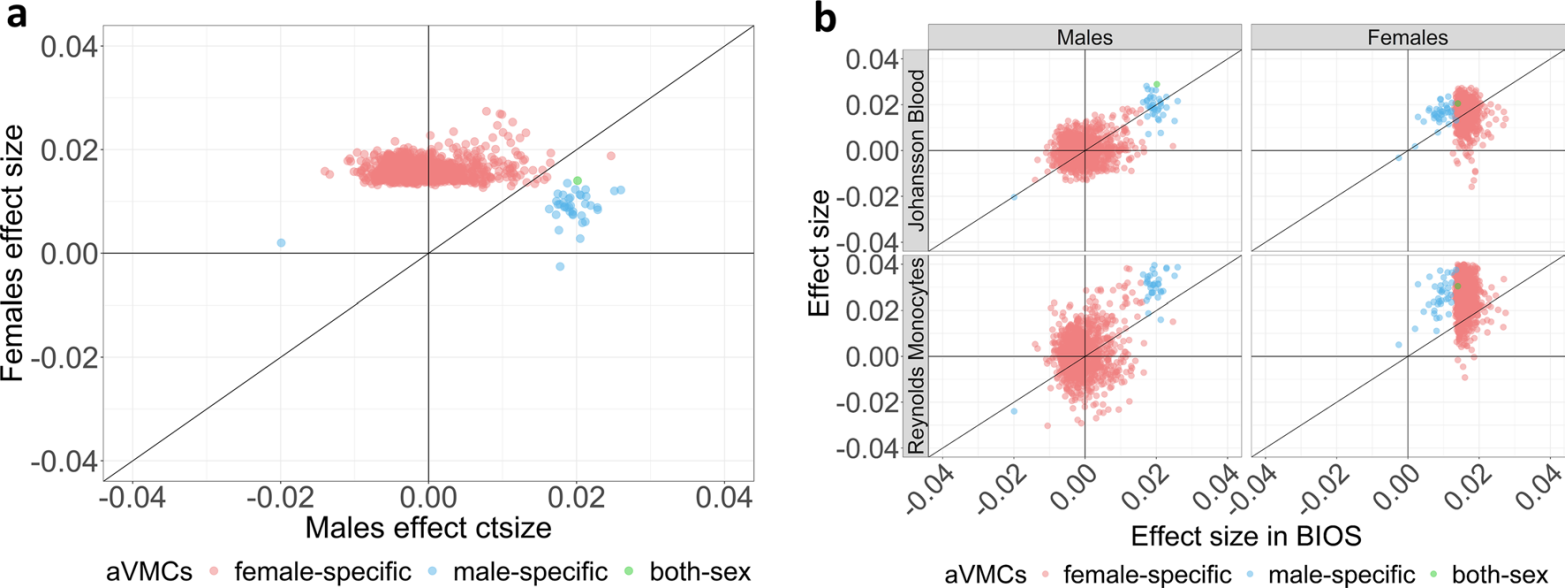
**a** Scatter plot of effect sizes for aVMCs for females and males in the discovery data set (BIOS Blood). Mean effect size of male-specific aVMCs in females were 0.4 times lower than that in males. **b** Scatter plot of aVMCs effect sizes in the discovery data sets and the Johansson Blood and Reynolds Monocytes replication datasets. Female-specific aVMCs, male-specific aVMCs and aVMCs statistically significant in both sexes are colored by red, blue and green, respectively.* aVMCs*, age-related variably methylated CpGs

Next, we used our discovery data set to identify CpGs whose variability was associated with age (aVMCs) by fitting the dispersion sub-model of DGLM. In contrast to aDMCs, aVMCs were more common in females (*n* = 1098) than males (*n* = 39) with only one overlapping CpGs (P_*bonf*_ < 0.05, Table 2, Additional file 2: Tables S5 and S6). In line with the findings for aDMCs, male-specific aVMCs had an effect size in females that was approximately diluted by a factor 2 (the mean effect sizes were 0.02 and 0.01 in males and females, respectively; Fig. 4a). Unlike aDMCs, aVMCs replicated surprisingly well in both the external whole blood and the external monocyte dataset for both sexes (> 90%; Table 2 and Fig. 4b), which may be partly explained by earlier observations that aVMCs are less dependent on cell type composition of blood [10, 13, 45]. In total, 987 females aVMCs (distributed across 658 distinct loci) and 37 males aVMCs (all represented distinct loci) replicated in both external data sets (Table 2, Additional file 1: Fig. S5, Additional file 2: Tables S5 and S6). Of note, all aVMCs were associated with an increase in variance with age except one (cg25871420), which decreased in variance with age specifically in males, also in both replication data sets (Table 2, Additional file 1: Fig. S4, Additional file 2: Table S5).

**Table 2:** Number of age-related variably methylated CpGs (aVMCs) in the discovery and replication stage

	Males			Females		
	*N*	aVMCs (loss/gain)	Replication rate	*N*	aVMCs (loss/gain)	Replication rate
*Discovery*						
BIOS Blood	1688	39 (1/38)	–	2343	1098 (0/1098)	–
*Replication*						
Johansson Blood	341	37 (1/36)	95%	388	1030 (0/1030)	94%
Reynolds Monocytes	583	39 (1/38)	100%	603	1035 (0/1035)	94%
Doubly replicated	–	37 (1/36)	95%	–	987 (0/987)	90%

### Annotation of X-linked aDMCs and aVMCs

**Fig. 5:**
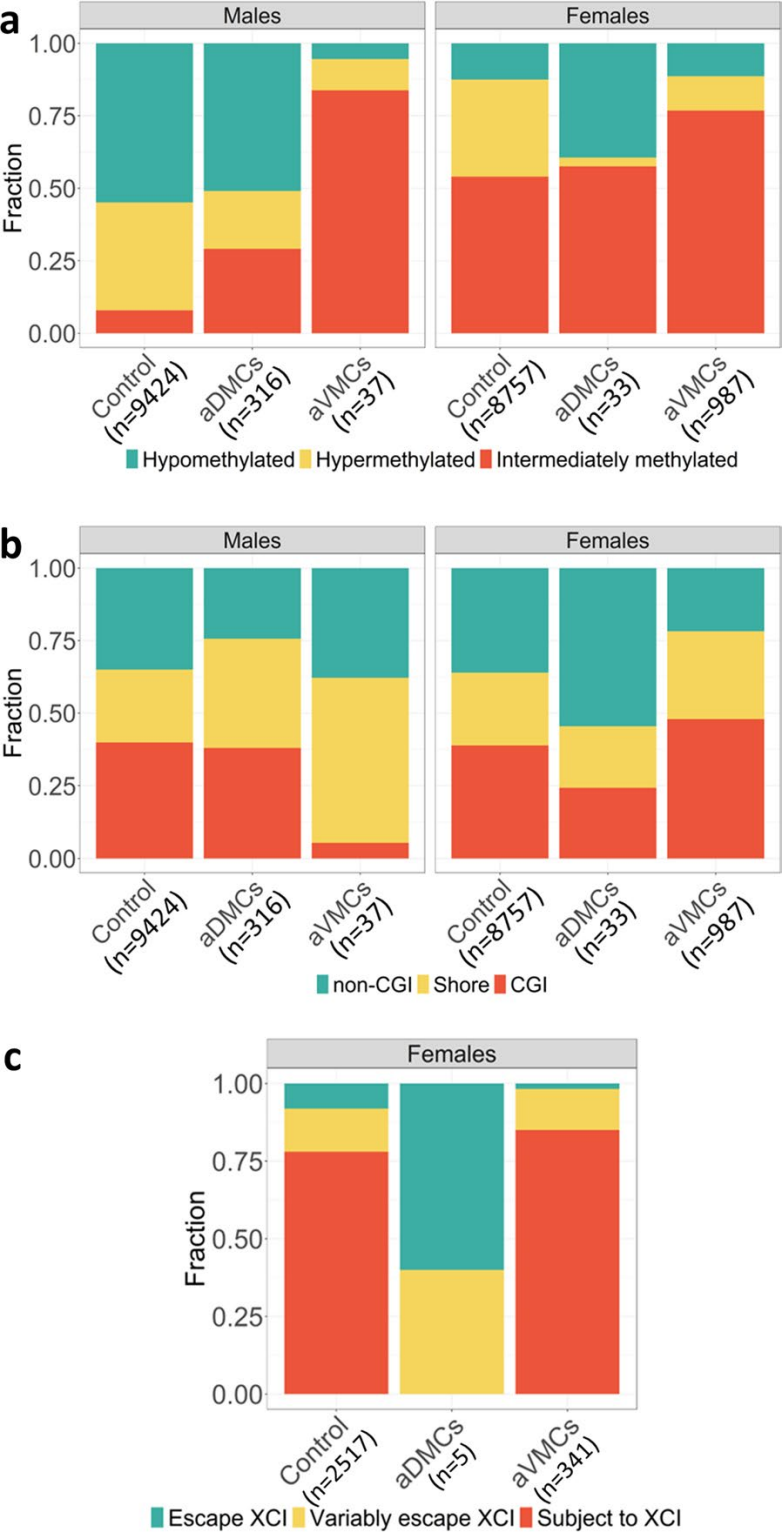
Annotation of aDMCs and aVMCs in males and females. **a** Categories of mean methylation level of aDMCs and aVMCs in males and females (intermediate methylated: 0.25 < *β* < 0.7, hypomethylated: *β* < 0.25, hypermethylated: *β* > 0.7). **b** Fraction of aDMCs and aVMCs in CGI-related features (CGI, CGI shore, and non-CGI). **c** XCI status annotation of aDMCs and aVMCs in females. Annotation was based on colocalization (< 2kb) to TSS categorized as Subject to XCI, Escape XCI and Variably escape XCI. *aDMCs*—age-related differentially methylated CpGs, *aVMCs*—age-related variably methylated CpGs, *CGI*—CpG island, *XCI*—X-chromosome inactivation

The striking contrast in the occurrence of aDMCs and aVMCs in males and females indicated a differential involvement of Xa and Xi in the two types of age-related DNA methylation differences. A first feature associated with Xi is hypermethylation resulting in a predominance of intermediate DNA methylation levels in females (Fig. 1). Compared to control CpGs, both aDMCs (P = 1 × 10^–6^) and aVMCs (P = 2 × 10^–16^) in females were preferentially intermediately methylated (Fig. 5a, Table S2). Secondly, it is known that in particular CpG island (CGI) methylation is involved in XCI [6]. Nevertheless, aDMCs were depleted at CGIs in females and were even more common in non-CGI regions (P = 0.08, Fig. 5b, Table S2). However, aVMCs did preferentially occur at CGIs in females (P = 2 × 10^–16^) while they were depleted in CGIs in males (P = 8 × 10^–7^). Finally, we investigated the XCI-status of aDMCs and aVMCs in females. This analysis was restricted to those that were within 2kb of a TSS of a gene and had known XCI status to ensure validity of the XCI-status prediction (Fig. 5c, Table S2). Female aDMCs all occurred outside regions subject to XCI (3 in escape and 2 in variably escape regions; P = 2 × 10^–3^). In contrast, the large majority (85%) of annotated female aVMCs occurred in regions subject to XCI (P = 1 × 10^–4^), in line with their enrichment at intermediately methylated regions and CGIs.

### Associations with X-linked gene expression

To explore whether the methylation level of aDMCs and aVMCs was associated with gene expression, we investigated the relationship between DNA methylation and 512 X-linked genes using a subset of 3131 individuals (1794/1337 females/males) from the discovery cohort for whom gene expression data were available in addition to DNA methylation data.

**Fig. 6:**
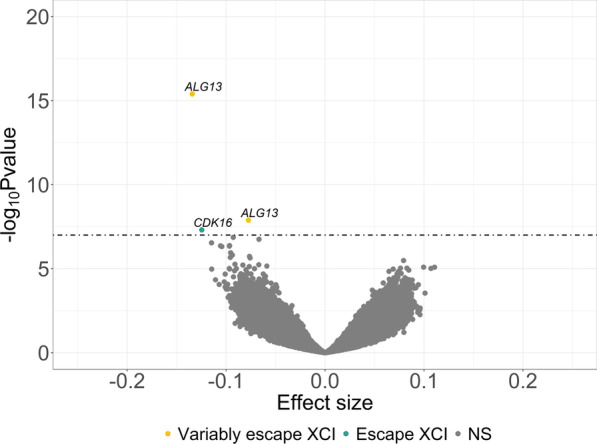
Volcano plot showing association between female-specific aVMCs methylation and X-chromosome gene expression in females (*n* = 1794, range 18–85 years). *aVMCs*—age-related variably methylated CpGs

No associations with gene expression were observed for aVMCs in males and aDMCs in females. For 66 male-specific aDMCs, an association was observed with the expression of 19 X-linked genes (Additional file 3: Table S7). For the majority of associations, the CpG-gene distance was > 100kb (*n* = 82). The genes included several immune related genes (e.g., *FRMPD3 and CXCR3*) in support of the interpretation that the Xa-specific aDMCs observed in males are more likely to depend on cell composition. For aVMCs, we only found that 3 female-specific CpGs associated with the expression of 2 X-linked genes, namely *ALG13* (2 CpGs: one in gene body and one > 1MB downstream; Fig. 6, Additional file 3: Table S8) and *CDK16* (CpG in gene body; Fig. 6, Additional file 3: Table S8). Neither aDMC- nor aVMC-associated genes were enriched for specific biological processes.

## Discussion

We report on a systematic analysis of age-related differences in X-chromosome methylation at the level of both differences in mean (age-related differentially methylated CpGs, aDMCs) and differences in variability (age-related variably methylated CpGs, aVMCs). We observed striking contrasts between the two types of age-related differences. aVMCs were common in females, rare in males and highly consistent across replication cohorts including in samples of purified monocytes. This suggests that the occurrence of X-linked aVMCs may be a cell-intrinsic phenomenon in line with previous reports for autosomal aVMCs [10, 13, 45]. More commonly studied aDMCs, however, were rare in females and common in males, showed a poor replication rate, in particular in purified monocytes, indicating that X-linked aDMCs may frequently be driven by changes in blood cell composition with age. Further analysis supported the interpretation that aVMCs preferentially occur in regions subject to XCI on the inactive X, as they were enriched in CGIs and regions subject to XCI. Taken together, our data imply that DNA methylation marks involved in XCI commonly accumulate variability with age, hence suggesting that a gradual waning of epigenetic control at the inactive X may be a feature of female aging.

While aVMCs have previously not been reported for chromosome X, three previous studies reported on X-linked aDMCs. However, there was a great discrepancy between the aDMCs reported by these studies. First, McCartney et al. analyzed one discovery cohort and one replication data set and reported only 5 sex-independent and 6 sex-dependent X-linked aDMCs using a linear regression approach that included both sexes and an interaction term between sex and age [9]. We observed one of the sex-independent aDMCs (cg25140188) and two of the sex-dependent aDMCs (cg20202246 and cg08814148) in our analysis (Additional file 2: Table S3). Next, Li et al. analyzed two discovery cohorts and found 559 and 1378 male-specific, and 1367 and 1148 female-specific aDMCs. Surprisingly, the overlap between findings of the two discovery cohorts was limited (34%-38%; Additional file 1: Fig. S6) and the replication rate in an external cohort was low (5–7%; Additional file 1: Fig. S6) [7]. We also found limited overlap between aDMCs from the two discovery cohorts and our replicated aDMCs (0.2%–3%; Additional file 1: Fig. S6). The high frequency of aDMCs in females as compared with males in the two discovery cohorts of Li et al. is at odds with the a priori expectation that the analysis of X methylation will have less power in females than males, because Xa-specific DNA methylation differences will be diluted by the presence of an additional Xi and vice versa, while males carry a single Xa only (Fig. 3). Finally, Kananen et al. analyzed 5 studies and reported aDMCs that were observed in at least 2 out of 5 studies [8]. Their findings overlapped to a higher degree with our replicated aDMCs (7%–17%, Additional file 1: Fig. S6), and the overlap substantially increased when we applied a similar replication criterion to the Kananen aDMC set as in our current study, namely being a significant aDMC in 3 studies instead of 2 (30%–32%, Additional file 1: Fig. S6). All in all, our aDMC results are more similar to that reported by Kananen et al. [8] than that reported by Li et al. [7]. Remaining differences between our study and the previous ones may be explained by differences in the study design and data analysis. Our total samples size (3334 females and 2612 males in discovery and replication cohorts) was substantially higher than that analyzed by Li (488 females and 488 males in discovery and replication cohorts) and Kananen (1191 females and 1240 males across 5 public datasets) [7, 8]. Moreover, among our replication cohort was a study based on purified monocytes instead of whole blood samples, rendering our results substantially less sensitive to blood cell type composition. Furthermore, we were more conservative in calling aDMCs. To correct for multiple testing, we applied Bonferroni correction in our discovery cohort and the false discovery rate for replication, whereas the previous studies used the more liberal false discovery rate for all analyses. In addition, we corrected for statistical inflation of test statistics (Additional file 1: Fig. S7), a common problem in genomics studies which induces false positive findings [46]. Finally, we adhere to a strict replication scheme and report only those aDMCs that were replicated in all cohorts. Moreover, it should be noted that in population studies like ours, findings in females are the average of Xa and Xi, in contrast to findings in males which can only stem from the Xa. This reduces the sensitivity to detect effects in females, but also the ability to definitely assign findings to either Xa or Xi. Nevertheless, our set of replicated aDMCs may be a robust starting point for further studies.

We observed that X-linked aVMCs are common in females and are associated with XCI. A main remaining question is whether aVMCs have functional relevance and how they can relate to the aging process in females. Several aVMCs mapped to genes implicated in age-related, female-specific diseases. For example, one aVMCs (cg07876586) mapped to the progesterone receptor membrane component 1 (*PGRMC1*), a gene associated with female-specific cancer types such as breast cancer [47] and ovarian cancer [48] (Additional file 1: Fig. S4, Additional file 2: Table S6). Another aVMC (cg23501813) mapped to Bruton’s tyrosine kinase (BTK) which is a key mediator of B cell receptor signaling and is linked to age-related autoimmune diseases common in females such as rheumatoid arthritis (RA) [49] and systemic lupus erythematosus (SLE) [50] (Additional file 2: Table S6). However, only for two X-linked genes we observed an association between aVMC methylation and expression. The genes included *ALG13*, which is known to variably escape XCI [51], and *CDK16*, which is annotated as a gene escaping XCI. This low yield may be explained in at least two ways. First, DNA methylation at aVMCs simply has little effect on gene expression. This would contrast with the assumed key role of DNA methylation in establishing and maintaining XCI. However, XCI involves multiple levels of epigenetic repression beyond DNA methylation (e.g., histone modifications). These other levels may not or be less affected with age. However, it could also be the case that age-related differences in DNA methylation levels may not have reached a putative threshold necessary for loss of XCI considering that most participants in our study were relatively young (mean age of 51 years with a range from 18 to 85 years). The latter links to a second potential explanation. It may be hypothesized that only when aVMCs reach extreme DNA methylation levels, for example in very old age, de-repression of regions normally subject to XCI occurs in a detectable way. Testing this hypothesis will require larger data sets and such analyses will have to consider the fact that aVMCs result from stochastic phenomena whose occurrence in frequency and location may be individual-specific.

Aging is also associated with loss of X chromosome (LOX) although its frequency may be low [52, 53]. As the phenomenon is thought to predominantly affect the Xi, one would expect LOX to increase the number of female-specific aDMCs whose methylation should predominantly decrease with age since the highly methylated Xi is lost and only the lowly methylated Xa remains. However, we observed many more male-specific aDMCs than female-specific aDMCs and, among females, the number of loss and gain aDMCs were similar (41 and 39, respectively; Table 1). A second age-related phenomenon is the skewing of XCI toward one parental X chromosome with age [54]. It remains unclear how skewing might affect the occurrence of aDMCs and aVMCs on the X chromosome in females relative to males. Our study did not allow us to assess the occurrence of LOX or skewing in the cohorts analyzed but it can be expected that these phenomena do not affect our overall conclusions.

## Conclusions

Our analysis revealed that age-related DNA methylation changes in females, likely affecting the inactive X chromosome, are dominated by the accumulation of variability instead of commonly studied differences in mean. This implies that the epigenetic control of XCI may gradually wane with age. The putative functional impact of this phenomenon to female aging may need to be studied in aged populations.

### Supplementary Information

**Additional file 1**. **Table S1** Characteristics of cohorts used in present study. **Table S2** The number and percentage of aDMCs and aVMCs in XCI related annotation features. **Fig. S1** Comparison of aDMCs effect size in both sex between DGLM and limma. **Fig. S2** Scatter plot of standard error for aDMCs effect size observed in males and females in the discovery data set (BIOS Blood). **Fig. S3** UpSet plot showing number of statistically significant overlapping aDMCs in males (a) and females (b) between discovery cohort (BIOS blood) and replication cohort (Johansson Blood and Reynolds Monocytes). Horizontal bars on the lower left corner of figure represent the number of aDMCs detected in the discovery data set and the subsets replicated in the two external data sets. The number of shared aDMCs between data sets represented as vertical bars and the data-sets involved with dots connected with lines. The vertical orange bar represents the number of aDMCs observed in all 3 data sets and are considered replicated aDMCs. Abbreviations: aDMCs age-related differentially methylated CpGs. **Fig. S4** Examples of replicated aDMCs and aVMCs in females and males. Left scatter plot showing aDMCs that change in average DNA methylation with age. The aDMCs methylation (mean effects: middle line indicated) increased or decreased with age. Right scatter plot showing aVMCs methylation that change in variance with age. The aVMCs methylation variance (dispersion effect: extra two lines indicated) increased or decreased with age. DNA methylation value were rank-inverse normal transformed (y-axis). Abbreviations: aDMCs age-related differentially methylated CpGs, aVMCs age-related variably methylated CpGs. The CpGs were selected from the top 10 lowest p-values per category. **Fig. S5** UpSet plot showing number of statistically significant overlapping aVMCs in males (a) and females (b) between discovery cohort (BIOS blood) and replication cohort (Johansson Blood and Reynolds Monocytes). Horizontal bars on the lower left corner of figure represent the number of aVMCs detected in the discovery data set and the subsets replicated in the two external data sets. The number of shared aVMCs between data sets represented as vertical bars and the data-sets involved with dots connected with lines. The vertical orange bar represents the number of aVMCs observed in all 3 data sets and are considered replicated aVMCs. Abbreviations: aVMCs age-related variably methylated CpGs. **Fig. S6** Comparison of replicated aDMCs catalogue in our study with previous study. a Li et al. vs Kananen et al. b Li et al vs Our study. c Kananen et al vs Our study. **Fig. S7** Histogram of test statistics for aDMCs in males (a) and females (b) separately. The black line represents the overall fit, the red line is the fit of empirical null distribution with estimated mean and variance. The green and blue lines represent the proportion of true positively and true negatively associations.

**Additional file 2. Table S3**. The list of age-related differentially methylated CpGs (aDMCs) in males. **Table S4** The list of age-related differentially methylated CpGs (aDMCs) in females. **Table S5** The list of age-related variably methylated CpGs (aVMCs) in males. **Table S6** The list of age-related variably methylated CpGs (aVMCs) in females.

**Additional file 3. Table S7** The association between male-specific aDMCs and X-chromosome gene expression in males (n=1337). **Table S8** The association between female specific aVMCs and X-chromosome gene expression in females (n=1794).

## Data Availability

The BIOS whole blood samples are available from the European Genome-Phenome Archive (EGAC00001000277). Scripts for the analyses in this manuscript are available at GitHub [https://github.com/YunfengLUMC/Epigenetic_variability_chrX] under an open source MIT license. All external datasets used in this study are publicly available from Gene Expression Omnibus (GEO) under accession numbers GSE87571 [32] and GSE56046 [33].
