# Supplementary material for: The inactive X chromosome accumulates widespread epigenetic variability with age
Source: Clin Epigenetics. 2023 Aug 25;15:135. doi: 10.1186/s13148-023-01549-y (PMC10464315; doi:10.1186/s13148-023-01549-y)

**Table S1 Characteristics of cohorts used in present study.**

| Cohort | N | |  | Age (years) | | Accession |
| --- | --- | --- | --- | --- | --- | --- |
|  | Males  (DNAm/RNA-seq) | Females  (DNAm/RNA-seq) |  | Mean | Range |  |
| **Discovery**  **(BIOS Blood)** |  |  |  |  |  |  |
| CODAM | 86/80 | 74/67 |  | 65 | 50-79 | EGAC00001000277 |
| LL | 313/282 | 427/374 |  | 46 | 18-81 |  |
| LLS | 344/300 | 375/326 |  | 58 | 30-79 |  |
| NTR | 485/287 | 933/567 |  | 37 | 18-79 |  |
| PAN | 107/94 | 70/61 |  | 62 | 37-87 |  |
| RS | 353/294 | 464/399 |  | 68 | 38-87 |  |
| **Replication** |  |  |  |  |  |  |
| Johansson Blood | 341/0 | 388/0 |  | 47 | 14-94 | GSE87571 |
| Reynolds Monocytes | 583/0 | 603/0 |  | 60 | 44-83 | GSE56046 |

DNAm: how many samples per cohort only DNA methylation data are available. RNA-seq: how many samples per cohort RNA-seq data and DNA methylation data are both available.

**Table S2 The number and percentage of aDMCs and aVMCs in XCI related annotation features.**

|  | **Males** | | |  | **Females** | | |
| --- | --- | --- | --- | --- | --- | --- | --- |
|  | **Control CpGs*** | **aDMCs** | **aVMCs** |  | **Control CpGs*** | **aDMCs** | **aVMCs** |
| **DNA methylation level** |  |  |  |  |  |  |  |
| Hypomethylated (β<0.25) | 5172 (55%) | 161 (51%) | 2 (5%) |  | 1095 (12%) | 13 (39%) | 112 (11%) |
| Hypermethylated (β>0.7) | 3506 (37%) | 63 (20%) | 4 (11%) |  | 2935 (34%) | 1 (3%) | 117 (12%) |
| Intermediately methylated (0.25<β<0.7) | 747 (8%) | 92 (29%) | 31 (84%) |  | 4728 (54%) | 19 (58%) | 758 (77%) |
| Total | 9424 (100%) | 316 (100%) | 37 (100%) |  | 8757 (100%) | 33 (100%) | 987 (100%) |
| P-value |  | 2x10^-16^ | 2x10^-16^ |  |  | 1x10^-6^ | 2x10^-16^ |
|  |  |  |  |  |  |  |  |
| **CGI feature** |  |  |  |  |  |  |  |
| Non-CGI | 3300 (35%) | 77 (24%) | 14 (38%) |  | 3159 (36%) | 18 (55%) | 215 (22%) |
| shore | 2366 (25%) | 119 (38%) | 21 (57%) |  | 2199 (25%) | 7 (21%) | 299 (30%) |
| CGI | 3759 (40%) | 120 (38%) | 2 (5%) |  | 3400 (39%) | 8 (24%) | 473 (48%) |
| Total | 9424 (100%) | 316 (100%) | 37 (100%) |  | 8757 (100%) | 33 (100%) | 987 (100%) |
| P-value |  | 5x10^-7^ | 8x10^-7^ |  |  | 0.08 | 2x10^-16^ |
|  |  |  |  |  |  |  |  |
| **XCI status** |  |  |  |  |  |  |  |
| Escape XCI | - | - | - |  | 204 (8%) | 3 (60%) | 6 (2%) |
| Variably Escape XCI | - | - | - |  | 349 (14%) | 2 (40%) | 45 (13%) |
| Subject to XCI | - | - | - |  | 1964 (78%) | 0 (0%) | 290 (85%) |
| Total | - | - | - |  | 2517 (100%) | 5 (100%) | 341 (100%) |
| P-value | - |  |  |  |  | 2x10^-3^ | 1x10^-4^ |

Control CpGs*: all CpGs on X-chromosome excluding those identified as aDMCs or aVMCs. Only CpGs within 2 kb from a TSS were annotated when calling XCI status. P-values were obtained using a Chi-square test or, when expected cell counts were lower than 5, the Fisher’s exact test. *Abbreviations*: *XCI, X-chromosome inactivation*

**Figure S1 Comparison of aDMCs effect size in both sex between DGLM and limma.**


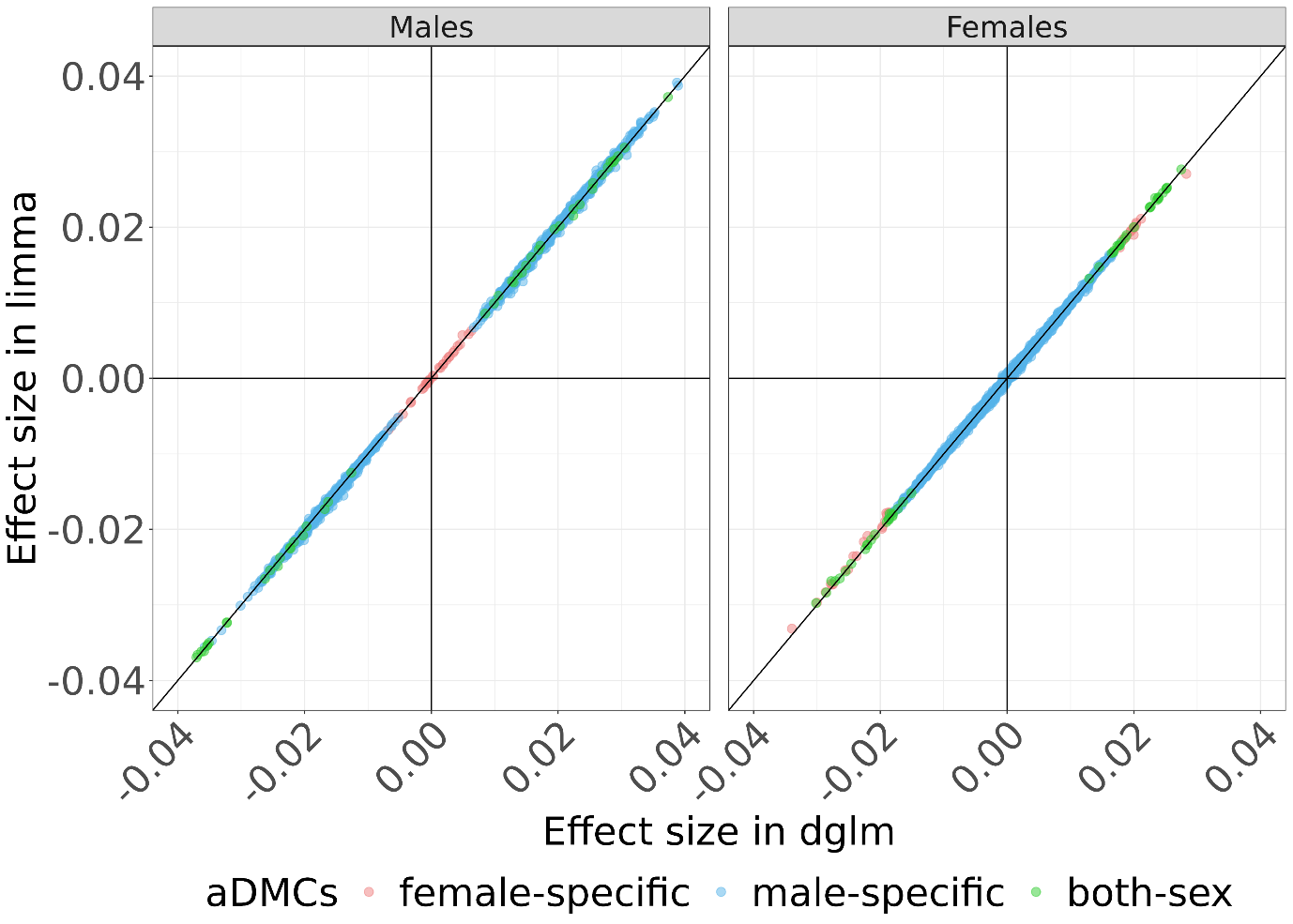


**Figure S2 Scatter plot of standard error for aDMCs effect size observed in males and females in the discovery data set (BIOS Blood).**





**Figure S3** **UpSet plot showing number of statistically significant overlapping aDMCs in males (a) and females (b) between discovery cohort (BIOS blood) and replication cohort (Johansson Blood and Reynolds Monocytes).** Horizontal bars on the lower left corner of figure represent the number of aDMCs detected in the discovery data set and the subsets replicated in the two external data sets. The number of shared aDMCs between data sets represented as vertical bars and the data-sets involved with dots connected with lines. The vertical orange bar represents the number of aDMCs observed in all 3 data sets and are considered replicated aDMCs. *Abbreviations*: *aDMCs* age-related differentially methylated CpGs

**
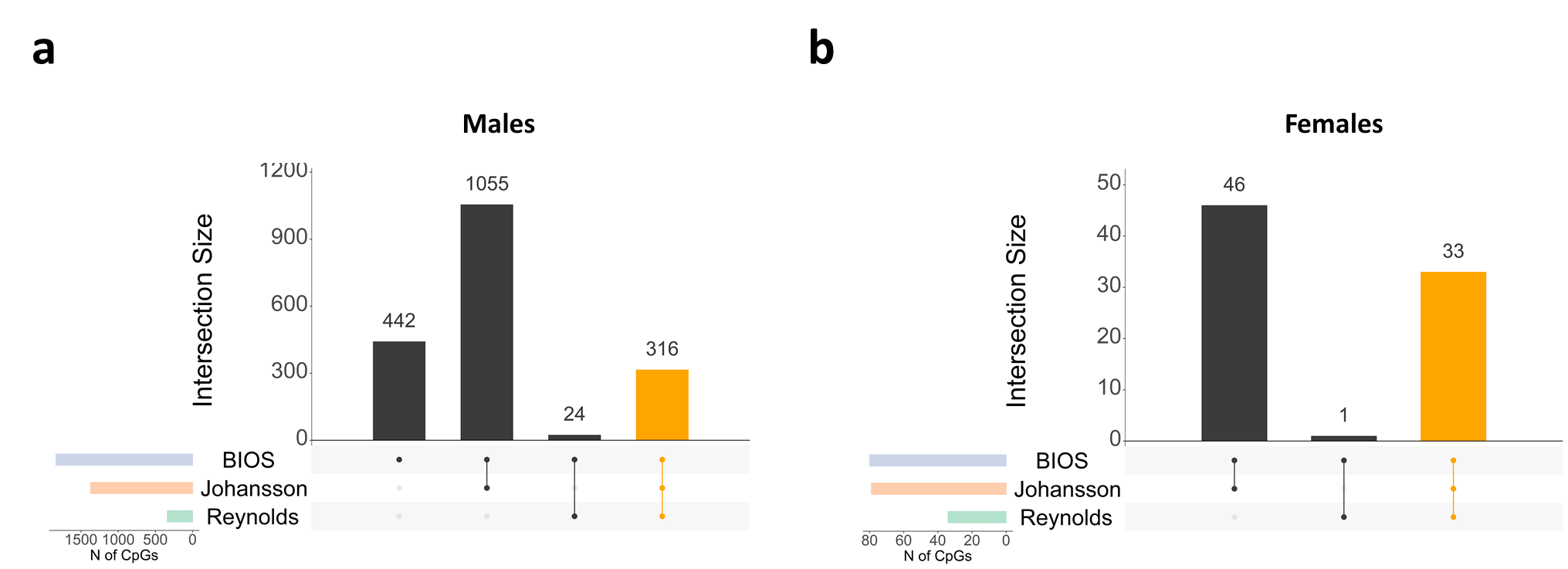
**

**Figure S4 Examples of replicated aDMCs and aVMCs in females and males.** Left scatter plot showing aDMCs that change in average DNA methylation with age. The aDMCs methylation (mean effects: middle line indicated) increased or decreased with age. Right scatter plot showing aVMCs methylation that change in variance with age. The aVMCs methylation variance (dispersion effect: extra two lines indicated) increased or decreased with age. DNA methylation value were rank-inverse normal transformed (y-axis). *Abbreviations*: *aDMCs* age-related differentially methylated CpGs, *aVMCs* age-related variably methylated CpGs. The CpGs were selected from the top 10 lowest p-values per category.





**Figure S5 UpSet plot showing number of statistically significant overlapping aVMCs in males (a) and females (b) between discovery cohort (BIOS blood) and replication cohort (Johansson Blood and Reynolds Monocytes).** Horizontal bars on the lower left corner of figure represent the number of aVMCs detected in the discovery data set and the subsets replicated in the two external data sets. The number of shared aVMCs between data sets represented as vertical bars and the data-sets involved with dots connected with lines. The vertical orange bar represents the number of aVMCs observed in all 3 data sets and are considered replicated aVMCs. *Abbreviations*: *aVMCs* age-related variably methylated CpGs


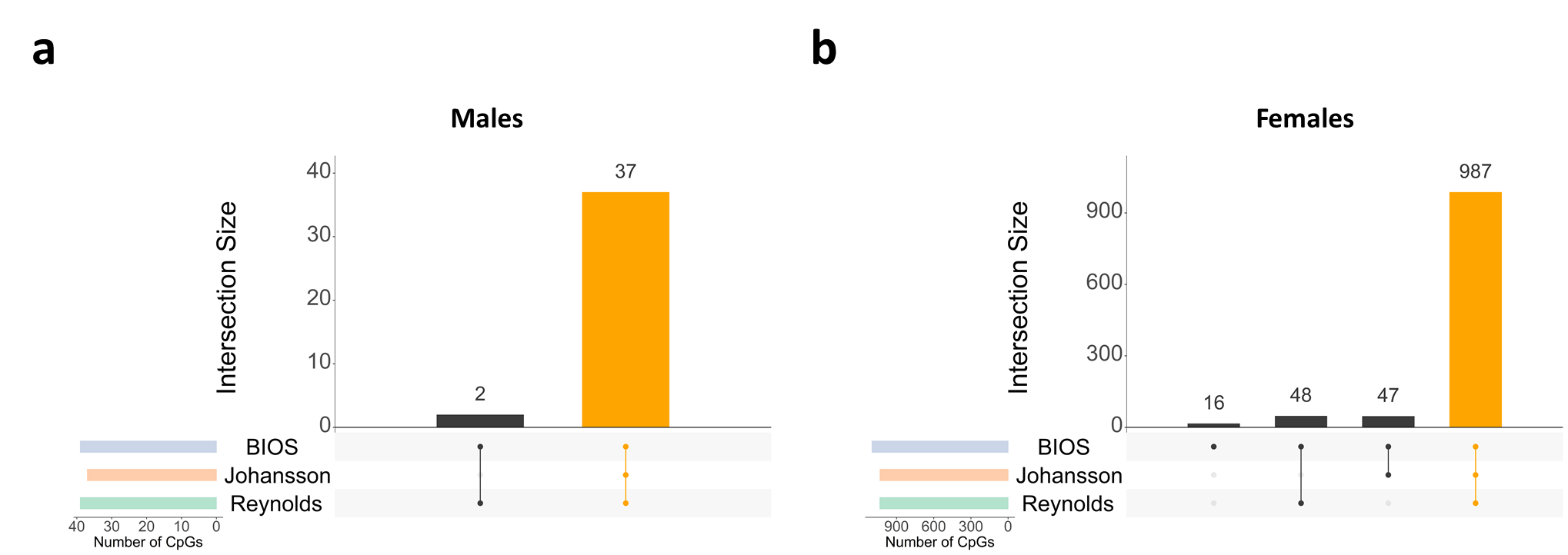


**Figure S6 Comparison of replicated aDMCs catalogue in our study with previous study.** a *Li* et al. *vs* *Kananen* et al. b *Li* et al *vs* Our study. c *Kananen* et al *vs* Our study.


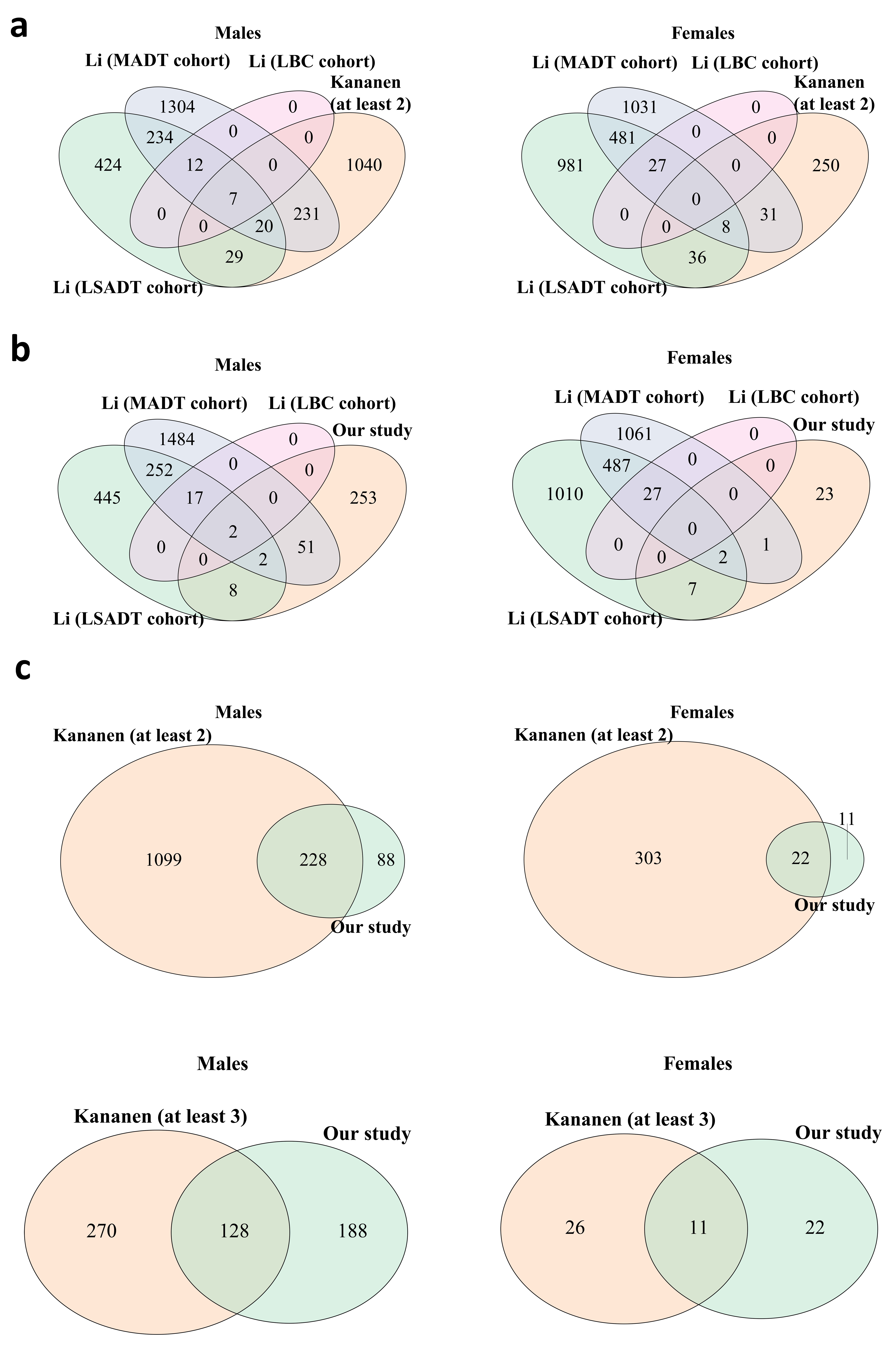


**Figure S7 Histogram of test statistics for aDMCs in males (a) and females (b) separately.** The black line represents the overall fit, the red line is the fit of empirical null distribution with estimated mean and variance. The green and blue lines represent the proportion of true positively and true negatively associations.


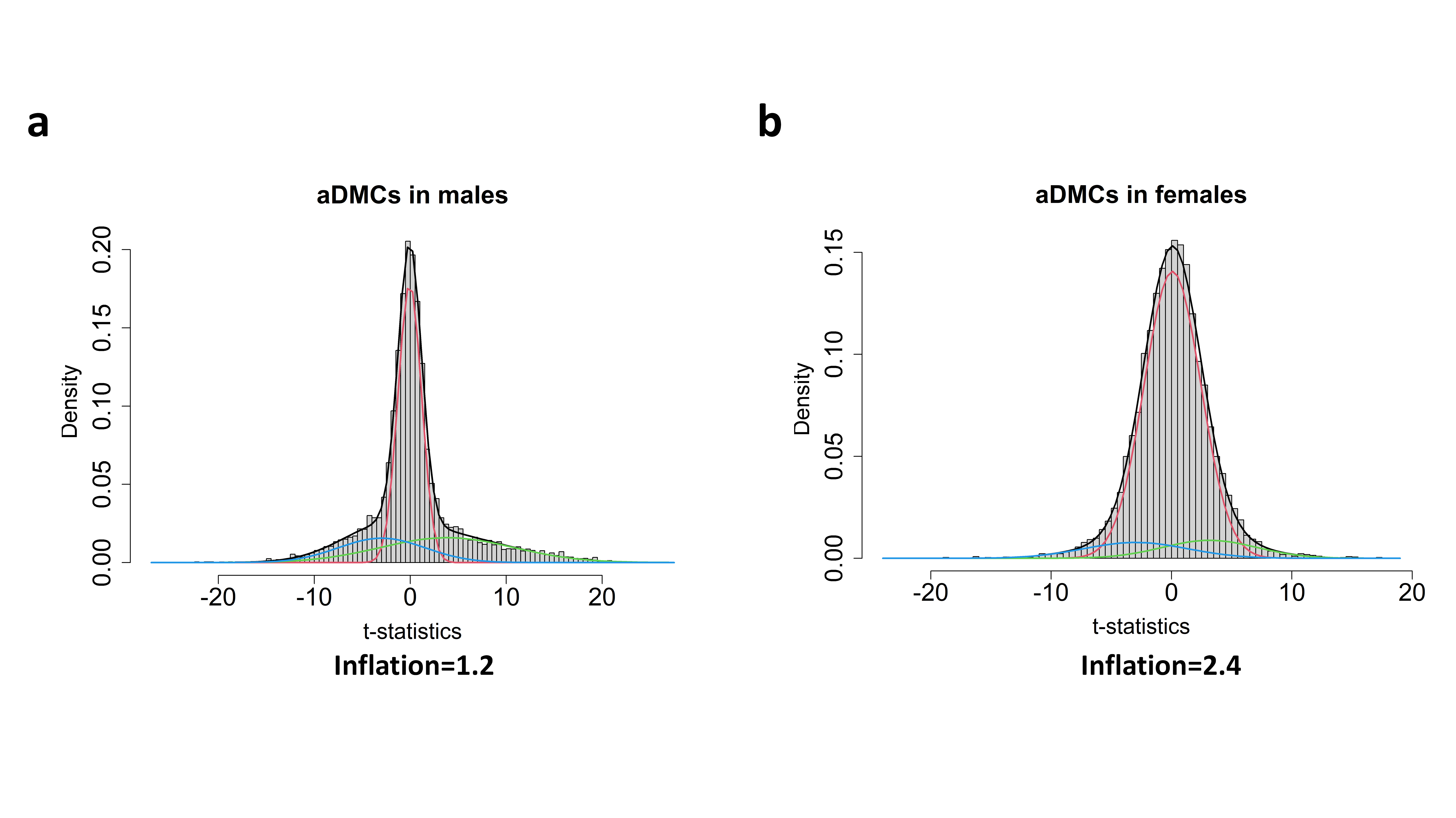

Supplement: Supplementary file 1 — Additional file 1. Table S1 Characteristics of cohorts used in present study. Table S2 The number and percentage of aDMCs and aVMCs in XCI related annotation features. Fig. S1 Comparison of aDMCs effect size in both sex between DGLM and limma. Fig. S2 Scatter plot of standard error for aDMCs effect size observed in males and females in the discovery data set (BIOS Blood). Fig. S3 UpSet plot showing number of statistically significant overlapping aDMCs in males (a) and females (b) between discovery cohort (BIOS blood) and replication cohort (Johansson Blood and Reynolds Monocytes). Horizontal bars on the lower left corner of figure represent the number of aDMCs detected in the discovery data set and the subsets replicated in the two external data sets. The number of shared aDMCs between data sets represented as vertical bars and the data-sets involved with dots connected with lines. The vertical orange bar represents the number of aDMCs observed in all 3 data sets and are considered replicated aDMCs. Abbreviations: aDMCs age-related differentially methylated CpGs. Fig. S4 Examples of replicated aDMCs and aVMCs in females and males. Left scatter plot showing aDMCs that change in average DNA methylation with age. The aDMCs methylation (mean effects: middle line indicated) increased or decreased with age. Right scatter plot showing aVMCs methylation that change in variance with age. The aVMCs methylation variance (dispersion effect: extra two lines indicated) increased or decreased with age. DNA methylation value were rank-inverse normal transformed (y-axis). Abbreviations: aDMCs age-related differentially methylated CpGs, aVMCs age-related variably methylated CpGs. The CpGs were selected from the top 10 lowest p-values per category. Fig. S5 UpSet plot showing number of statistically significant overlapping aVMCs in males (a) and females (b) between discovery cohort (BIOS blood) and replication cohort (Johansson Blood and Reynolds Monocytes). Horizontal bars on the lowe [file 13148_2023_1549_MOESM1_ESM.docx]
